# Role of Oxycodone Hydrochloride in Treating Radiotherapy-Related Pain

**DOI:** 10.1155/2020/7565962

**Published:** 2020-01-30

**Authors:** Yinxia Wang, Ligang Xing

**Affiliations:** Department of Radiation Oncology, The Shandong Cancer Hospital and Institute, Shandong First Medical University and Shandong Academy of Medical Sciences, Jinan, Shandong, China

## Abstract

Radiotherapy is commonly used to treat cancer patients. Besides the curable effect, radiotherapy also could relieve the pain of cancer patients. However, cancer pain is gradually alleviated about two weeks after radiotherapy. In addition, cancer patients who receive radiotherapy may also suffer from pain flare or radiotherapy-induced side effects such as radiation esophagitis, enteritis, and mucositis. Pain control is reported to be inadequate during the whole course of radiotherapy (before, during, and after radiotherapy), and quality of life is seriously affected. Hence, radiotherapy is suggested to be combined with analgesic drugs in clinical guidelines. Previous studies have shown that radiotherapy combined with oxycodone hydrochloride can effectively alleviate cancer pain. In this review, we firstly presented the necessity of analgesia during the whole course of radiotherapy. We also sketched the role of oxycodone hydrochloride in radiotherapy of bone metastases and radiotherapy-induced oral mucositis. Finally, we concluded that oxycodone hydrochloride shows good efficacy and tolerance and could be used for pain management before, during, and after radiotherapy.

## 1. Introduction

Radiotherapy is used as a curative or coadjuvant treatment for malignant tumors and plays an important role in the treatment of cancer. 60%∼70% of patients with cancer require radiotherapy in the clinic. Palliative radiotherapy is commonly used to treat patients with bone metastases to relieve pain-related symptoms. Previous studies showed that radiotherapy alone or combined with other treatment provides beneficial effect in cancer treatment for curative and palliative purposes [1]. Radiotherapy is used to treat approximately 80% of patients with head and neck cancer and undeniably achieves favorable disease control in the great majority of head and neck patients [2]. For other types of cancer, radiotherapy also has a clear benefit for both long-term survival and local recurrence rate [3–5].

However, radiotherapy slowly alleviates pain, and cancer pain is gradually alleviated about two weeks after radiotherapy. In addition, cancer patients who receive radiotherapy also suffer from radiotherapy-induced side effects such as radiation esophagitis, enteritis, and mucositis. It is reported that pain control is inadequate during the whole course of radiotherapy, and quality of life is seriously affected. Hence, it is stated in certain clinical guidelines that radiotherapy should be combined with analgesic drugs. Oxycodone hydrochloride is a semisynthetic opioid interacting with mu- and kappa-opioid receptors, with additional clinical benefit of oxycodone compared with morphine regarding visceral pain due to its characteristics on kappa-opioid receptors, and is primarily used to relieve moderate-to-severe pain. Previous studies have shown that radiotherapy combined with oxycodone hydrochloride can effectively alleviate cancer pain to improve treatment compliance and quality of life for cancer patients. This paper analyzed the status of pain control during radiotherapy and attempted to evaluate the role of oxycodone hydrochloride in the management of the pain during the whole course of radiotherapy.

## 2. Necessity of Analgesia during the Whole Course of Radiotherapy

Along with cancer pain, cancer patients receiving radiotherapy also have to experience radiotherapy-induced pain. Despite the types of effective analgesic methods available, the pain management for these patients remains poor. According to the latest research, the pain management was inadequate for almost 50% of cancer patients [6], among which 20% of cancer patients experienced inadequate management for surgery-, radiotherapy-, and chemotherapy-related pain [7]. In a survey of outpatients with bone metastases who received radiotherapy, the inadequate management of pain was also found to be common and has increased from year to year [8–10]. According to a recent study in 2016, the incidence of inadequate pain management was 33.3% among cancer outpatients undergoing palliative radiotherapy [11]. The reasons may be as follows: (1) there is a slow effect of radiotherapy on improving pain relief, as the cancer pain is gradually alleviated two weeks after radiotherapy; (2) there is inadequate pain control. The overall pain relief rate of radiotherapy was 59–88% for patients with bone metastases, and the complete pain remission rate was about 33% [12]; (3) patients experience pain flare and radiation-induced side effects; (4) radiotherapy provides short-term pain control; (5) chronic postradiotherapy pain occurs frequently [13]. Thus, during the whole course, radiotherapy should be combined with sustained adequate analgesics to control the pain to avoid the interruption of antitumor treatment and improve compliance and the life quality of cancer patients and their families.

### 2.1. Necessity of Analgesia before Radiotherapy

For cancer patients who require radiotherapy, almost a week is needed to initiate a series of preparations before radiotherapy, and when combined with the first 3 weeks after radiotherapy begins, approximately 3-4 weeks have passed before the patients experience pain relief. During this time, patients may suffer from more pain caused by tumor compression and invasion to an adjacent organ, nerve, or blood vessel, and the pain is often ignored by clinicians and thus is not well controlled. Poor pain control or even no management of pain will cause physical and mental suffering; meanwhile, it affects postural changes in radiotherapy, thus decreasing the compliance to radiotherapy in cancer patients. Therefore, analgesic treatment before radiotherapy can effectively alleviate the pain of patients during the waiting period and consequently improve compliance and the life quality of patients. Most of the nonsteroidal analgesics are fast-release tablets with a short half-life, which are suitable for treating mild pain; however, for treating moderate-to-severe cancer pain, opioid analgesics, such as oxycodone hydrochloride prolonged-release tablets, should be used.

Advanced cancer with bone metastases can cause pain, dysfunction, and pathological fractures, disturbing the daily lives of patients. Intense pain is the main symptom of bone metastases, which seriously affects the life quality and may interrupt the next step of antitumor therapy. Palliative radiotherapy can inhibit or kill tumor cells in bone tissue, reducing tumor volume, minimizing the pressure on the periosteum, and finally relieving pain [14]. Studies showed that pain was relieved with different degrees following palliative radiotherapy in 60–70% of patients with bone metastases, but complete pain remission was achieved in only approximately 25% of patients [15]. It was stated in the European Society for Medical Oncology (ESMO) clinical practice guidelines in 2018 that palliative radiotherapy and targeted therapy should be combined with analgesics for treating bone pain [16]. In addition, according to the consensus on clinical diagnosis and treatment of malignant bone metastases and bone-related diseases in 2014, palliative radiotherapy should be combined with analgesics to treat the pain caused by bone metastases [12].

### 2.2. Necessity of Analgesia during and after Radiotherapy

During the radiotherapy, sustained adequate analgesia is critical to prevent pain arising from clinical operation. During radiotherapy, postural changes or posture maintenance will further aggravate pain, leading to interruption or even withdrawal of treatment. Consequently, the rational use of analgesics in radiotherapy is necessary to avoid these situations and ensure the smooth performance of radiotherapy [17, 18].

Pain flare presents as a brief increase in systemic bone pain after palliative radiotherapy for bone metastases, especially following a single high dose [19], which is a common side effect of palliative radiotherapy. Pain flare means that pain scores (0–10 points) are increased by 2 points with the analgesic dose unchanged [20] or no rise in the pain score with an increased oral dose of morphine or other drugs by 25% [21]. Hird et al. found that the incidence of pain flare was 40% in 111 patients with bone metastases who received radiotherapy [21]. Doctors should pay attention to the treatment of pain flares because patients often have doubts about returning for radiotherapy when pain flares have been experienced, which can interfere with the remaining antitumor treatment plan. Therefore, oxycodone hydrochloride prolonged-release tablets are often preventively used in the clinic [17, 18] to reduce the incidence of pain flare during radiotherapy, decrease the patient's fear of radiotherapy, and improve patient compliance.

After radiotherapy, the patients almost also need the analgesics to help them to control the pain. For patients who suffer from cancer pain in the first 2 weeks after radiotherapy or who are not relieved completely by palliative radiotherapy, analgesics will help them to relieve pain effectively, increase compliance, and finally improve the life quality of patients and their families.

In addition, radiotherapy usually results in acute or chronic pain due to complications, such as radiation esophagitis, enteritis, and mucositis which all require analgesic treatment. Radiation esophagitis is a very common radiotherapy-associated complication in thoracic malignancies and is manifested clinically as dysphagia, odynophagia, and substernal discomfort or pain and usually occurs within 2-3 weeks after radiotherapy initiation, with healing within 3 weeks after completion of treatment [22]. Radiation enteritis can commonly occur following irradiation of abdominal or pelvic malignancies, resulting in abdominal pain, mucus or blood in stool, and diarrhoea. The symptoms generally disappear 2–6 weeks after the completion of radiotherapy [22] Radical radiation therapy is also a primary method to treat head and neck cancer. However, radiation-induced oral mucositis, a common radiotherapy-related complication for patients with head and neck cancer, appears 2-3 weeks after radiotherapy when the total radiation dose has reached 10∼30 Gy, remains at its peak for at least 2 weeks following the completion of radiotherapy (commonly 60–70 Gy), and then persists for up to 8 weeks [23]. The severity of radiation-induced oral mucositis is strongly associated with the dose, fraction size, radiation portals, fractionation, and the type of the ionizing radiation used [24]. The incidence of radiation-induced oral mucositis is 85% to 100% [25]; it reaches almost 100% for nasopharyngeal carcinoma patients undergoing intensity-modulated radiation therapy (IMRT) [26].

Due to the destruction of salivary glands during radiotherapy, reduced salivary secretion and altered salivary composition effectively eliminate the abilitiy of oral self-cleaning, which makes patients susceptible to bacterial and viral infections. In addition, radiation can directly damage the normal epithelial cells of the oral mucosa, and finally ulcers appear. Due to immune system damage after radiotherapy, cancer patients are more vulnerable to bacterial and viral invasion, which aggravates the injury of mucosal epithelium. Previous clinical treatments mainly consisted of oral cleaning, nutritional supplements, local mucosal recovery, and anti-infection treatment, while pain management was ignored. Consequently, eating is affected and leads to a weak immunity, and the treatment of radiotherapy and chemotherapy may be interrupted. Therefore, the use of analgesics is very important to treat radiation-induced oral mucositis in patients with nasopharyngeal carcinoma.

## 3. Role of Oxycodone Hydrochloride Prolonged-Release Tablets in Radiotherapy

Oxycodone, extracted from the alkaloid paramorphine, belongs to a potent pure opioid receptor agonist. Oxycodone is a relatively selective *µ*-opioid receptor agonist. Some animal studies have also shown that the antinociceptive effect of oxycodone is associated with activation of *κ*-opioid receptors [27, 28]. However, experiments have suggested that the antinociceptive effect of oxycodone can be inhabited by selective *µ*-opioid receptor antagonists, such as *β*-funaltrexamine (*β*-FNA), but not by selective *κ*-opioid receptor antagonists [29, 30].

**Figure 1:**
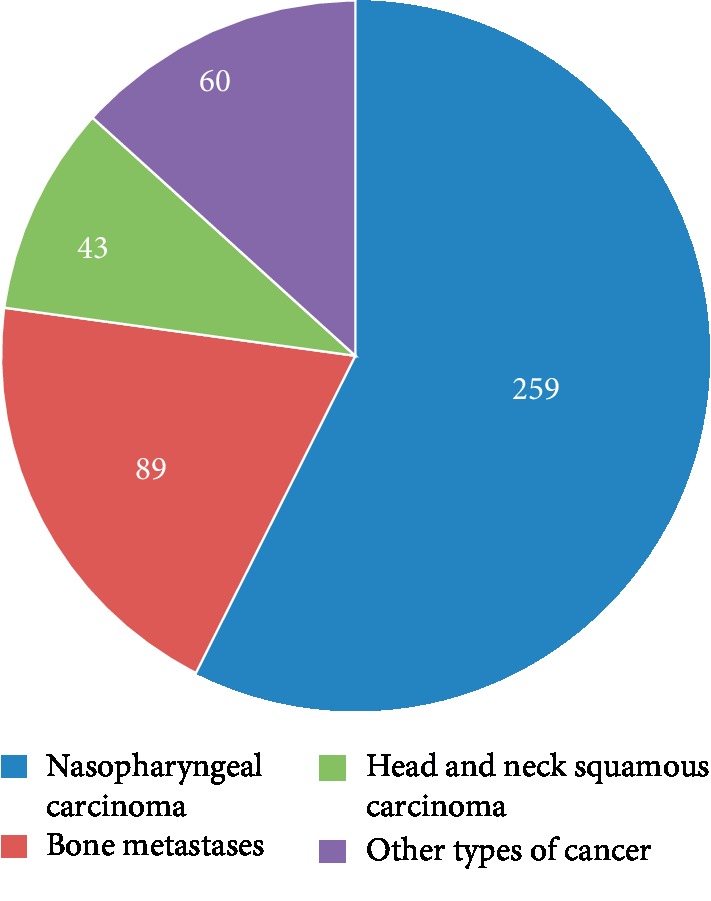
Distribution of the cases in 8 papers.

Oxycodone has intravenous, subcutaneous, intramuscular, and oral formulations, and oral formulations includes immediate-release (IR) tablets (for 4-hourly dosing) and sustained (controlled-release (CR)) release tablets (for 12-hourly dosing) [31]. The IR formulation allows for easy titration and is an important agent for the control of breakthrough pain. Due to individualization of the dose, oxycodone was titrated at the start of therapy and periodically thereafter to identify a stable dose associated with a favorable balance between analgesia and adverse effects [32]. For CR formulation, it is more important for the maintenance phase in cancer pain management. Furthermore, as recommended by the European Association of Palliative Care (EAPC), the IR and CR oral formulations of morphine, oxycodone, and hydromorphone can be used for dose titration, and for both types of formulation, should be supplemented with oral IR opioids given as needed. Oxycodone is used to relieve moderate-to-severe pain, especially for neuropathic pain and physical pain from malignant or nonmalignant diseases [33]. It was reported that oxycodone hydrochloride is more commonly used in the radiotherapy department to relieve cancer pain or radiotherapy-related pain and improve compliance and the life quality of cancer patients [17, 34]. Prof. Shang found that when compared with radiotherapy alone, the combined use of radiotherapy and oxycodone hydrochloride could achieve a lower pain intensity score and a higher pain relief rate for patients with advanced breast cancer [35]. In this study, we searched for 8 papers regarding the use of oxycodone hydrochloride in radiotherapy departments through the English and Chinese library, mainly in patients with bone metastases or nasopharyngeal carcinoma. Among these, 2 studies were in patients with bone metastases, 4 in nasopharyngeal carcinoma, 1 in head and neck carcinoma, and 1 in other types of cancer (Table 1, Figure 1).

### 3.1. Role of Oxycodone Hydrochloride in the Radiotherapy of Bone Metastases

Oxycodone hydrochloride has a strong affinity for opioid receptors in the brain and spinal cord, which can achieve an effective analgesic effect on bone metastases. Many clinicians have combined radiotherapy with oxycodone hydrochloride to treat moderate-to-severe pain caused by bone metastases of malignant tumors, achieving a combination of short-term and long-term analgesic effects. Hu et al. studied 47 cases of bone metastases with moderate-to-severe pain and found that in the first week when oxycodone hydrochloride was used alone, the overall pain relief rate was 95.7%. Since conventional radiotherapy had been performed in the second week, the pain remission rate was above 91.5% until the fourth week ended, and the pain remission rate reached 97.9% when the fifth week ended [36]. The local responses to radiotherapy consisted of dryness, pigmentation of skin, pruritus, and erythema. The adverse drug reactions included constipation (19.1%), nausea and vomiting (12.8%), urinary retention (4.2%), drowsiness (4.2%), and dizziness (14.9%), all of which were relieved by symptomatic treatment.

In a study by Yi et al. of 42 cancer patients with bone metastases, efficacy of oxycodone hydrochloride treatment alone was compared with that of the combined treatment with radiotherapy [17]. Radiotherapy was performed 3–7 days after oxycodone hydrochloride treatment. In the combined group, the pain relief rates in the 2nd, 4th, and 6th week were 80.9%, 85.7%, and 95.1%, respectively, which were significantly higher than those in the oxycodone alone group at the same time points (57.1%, 66.7%, and 71.4%, respectively, *P* < 0.05). The proportion of patients with an improved life quality in the combined group was significantly higher when compared to the oxycodone hydrochloride alone group (85.7% vs. 47.6%, *P* < 0.05). No significant difference was found in adverse reaction rate between the two groups (23.8% vs. 28.5%).

### 3.2. Role of Oxycodone Hydrochloride in Radiation-Induced Oral Mucositis

According to previous reports, strong opioid drugs such as oxycodone hydrochloride prolonged-release tablets administered during radiotherapy can effectively reduce the pain caused by radiation-induced oral mucositis for head and neck cancer patients. On the contrary, for patients without opioid drug treatment, the visual analog scale (VAS) pain score increased from 4 points to 7–9 points in the 3rd week of radiotherapy [41–43]. Compared with conventional therapy alone, Lin et al. found that after adding oxycodone hydrochloride to conventional therapy, the pain from radiation-induced oral mucositis was effectively controlled (96.7% vs. 33.4%), eating was significantly improved (83.4% vs 33.4%), and the proportion of body mass loss of more than 5% after oral ulcer healing was decreased (6.6% vs. 43.3%, *P* < 0.01) [38]. In addition, a similar study was performed by Wu et al. in 56 patients with nasopharyngeal carcinoma and discovered that after adding oxycodone hydrochloride to conventional treatment, pain was effectively relieved and the life quality of patients was significantly improved, whereas in the conventional treatment group, the VAS score was more than 7 points at 5–7 weeks after radiotherapy [39]. In the oxycodone group, the adverse reactions consisted of gastrointestinal reactions (25%), dizziness (7%), drowsiness (14%), constipation (21%), and other complications, which can be relieved by symptomatic treatment. There were no withdrawal symptoms when the medication was discontinued.

A randomized controlled trial by Yi Yu et al. [37] was performed to compare the efficiency of oxycodone hydrochloride prolonged-release tablets (*n* = 34) with that of fentanyl transdermal treatment (*n* = 32) in nasopharyngeal carcinoma patients affected by radiation-induced oral mucositis. It was shown that the pain was relieved in both groups, while the analgesic effect of the oxycodone group was significantly better than that of the fentanyl transdermal group in the 7th week of radiotherapy (VAS: 3.59 ± 0.28 vs. 4.42 ± 0.33, *P* < 0.05). The incidence of adverse reactions (constipation, nausea, vomiting, and dizziness) was not statistically significant between the two groups, and neither respiratory depression nor addiction occurred.

Another study found that the use of oxycodone hydrochloride in the early stage of radiation-induced oral mucositis achieved a better curative effect when compared with the use in the middle or late stage. Recently, a study by Hisamitsu et al. [40] investigated the efficiency of oxycodone in 43 head and neck cancer patients affected by radiation-induced oral mucositis for mild or moderate pain. The results found that when the cumulative dose reached 25–50 Gy, the VAS score in the mild pain group was significantly lower than that of the moderate pain group. The duration of regular diet was also longer in the mild group. With the increased radiation dose, a decrease in caloric intake will cause weight loss, but the rate of weight loss was significantly lower in the mild pain group than that in the moderate pain group. There were no significant differences between the two groups in terms of side effects, such as constipation, nausea, drowsiness, diarrhea, vomiting, pruritus, or dysuria. Serious adverse reactions, such as respiratory depression and mental disorder, did not occur in either group. Moreover, 102 radiation-induced oral mucositis patients with mild, moderate, and severe pain were treated with oxycodone hydrochloride in a study by Huang [26]. The results showed that patients in the mild pain group exhibited a more favorable effect in terms of VAS score, QOL (quality of life) score, and side effects. Hence, the initial use of oxycodone in mild pain arising from radiation-induced oral mucositis could avoid malnutrition, improve the life quality, and augment the effect of radiotherapy.

## 4. Discussion

Pain often existed in the whole course of radiotherapy. However, studies have shown that pain management is still inadequate for patients undergoing radiotherapy. The reason may arise from cancer pain in the waiting periods but overlooked easily, the slow effect of radiotherapy on improving pain relief, and the radiotherapy-related complications. Hence, clinicians should pay more attention to pain management. Timely, comprehensive, dynamic pain assessment and treatment should be performed before, during, and after radiotherapy. Combining radiotherapy with pain medication is crucial to achieve the full analgesic effect and further improve the life quality of patients and their families [26, 43]. Opioids including codeine, morphine, oxycodone, and hydromorphone, could be used in moderate-to-severe cancer pain management. Different formulations are administrated in various clinical settings. IR formulation is suitable for titration and breakthrough pain management, and CR formulations are more suitable for maintenance phase. Oral administration of opioids is usually preferable, whenever possible. For the patients who are unable to take oral medicines at some point in the course of their illness because of, for example, dysphagia, bowel obstruction, or vomiting, other routes of opioid administration are often needed. Oxycodone, a strong opioid interacting with mu- and kappa-opioid receptors, was used widely in patients with cancer pain. After reviewing relevant literature from China and other countries, we found that oxycodone hydrochloride can achieve good efficacy and tolerance for treating patients who are undergoing radiotherapy. Clinical guidelines suggested that palliative radiotherapy should be combined with analgesic drugs for cancer patients with bone metastases. The related studies have indicated that combining palliative radiotherapy with oxycodone hydrochloride can enhance the analgesic effect in cancer patients with bone metastases. Generally, due to overlooked cancer pain in the waiting periods and the slow effect of radiotherapy on improving pain relief, oxycodone is used before radiotherapy to relieve the cancer pain that patients suffer. Moreover, the use of oxycodone hydrochloride during radiotherapy can prevent the occurrence of pain flares and the pain from postural changes, avoiding an interruption in radiotherapy. Furthermore, oxycodone hydrochloride showed good analgesic effect for treating radiation-induced oral mucositis with a better analgesic effect initiated when the pain was mild.

In summary, radiotherapists should pay more attention to pain management in clinical practice and not only provide information of side effect of radiotherapy and pain management plan to patients and their family but also take appropriate treatment to relieve patients' pain and improve their QoL. As shown in this review, oxycodone hydrochloride shows good efficacy and tolerance profile and could be used for pain management before, during, and after radiotherapy.

## Figures and Tables

**Table 1:** Studies evaluating the analgesic efficacy of oxycodone hydrochloride in treating radiotherapy-related pain.

Study	Type of cancer	Sample size	Dose of oxycodone	Radiation dose	Administration time	Main outcomes	Side effects
Hu et al. [36]	Bone metastasis	47	20 mg, 40 mg, 60 mg	10–40 Gy	Administrated a week before radiotherapy until the fifth week of radiotherapy ended	The remission rate was 97.9% until the fifth week ended. The KPS score was increased by ≥20 in 23 patients (48.9%) and 10–20 in 17 patients (36.2%) when the treatment concluded	There were local responses to radiotherapy and adverse drug reactions, which were all relieved by symptomatic treatment

Yi [17]	Metastases of bone	Oxycodone alone (21): combined radiotherapy with oxycodone (21)	10 mg	NA	Administrated 3–7 days before radiotherapy till the sixth week of radiotherapy ended	The pain relief rate in the combined group was significantly higher than that in the oxycodone alone group in the 6th week of radiotherapy (95.1% vs. 71.4%, *P* < 0.05)	No significant difference was found between the two groups (23.8% vs. 28.5%, *P* > 0.05)

Yu et al. [37]	Nasopharyngeal carcinoma	Oxycodone group (34) and fentanyl group (32)	∼10 mg	70∼75 Gy	Administrated when the VAS score reached 4 or more in patients affected by radiation-induced oral mucositis	The analgesic effect in the oxycodone group was significantly better than that in the fentanyl group during 5th to 7th weeks of radiotherapy (VAS in 7th week of radiotherapy: 3.59 ± 0.28 vs. 4.42 ± 0.33, *P* < 0.05)	The incidence of adverse reactions (constipation, nausea, vomiting, and dizziness) was not statistically significant between the two groups (*P* > 0.05)

Huang and Zheng [26]	Nasopharyngeal carcinoma	102	∼10 mg	68∼70 Gy	Administered in the 2nd and 3rd week of radiotherapy	In the group given oxycodone for mild pain, the outcomes were more beneficial in terms of VAS score and QOL score compared with those treated for severe pain	The incidence of adverse reactions in the group treated with oxycodone for mild pain was significantly less than that in the group treated for severe pain (*P* < 0.05)
Lin and Wang [38]	Cancer patients affected by radiation-induced oral mucositis	Conventional therapy (30) and oxycodone combined with conventional therapy (30)	∼5 mg	NA	Administered when the pain from radiation-induced oral mucositis reached a moderate level	After adding oxycodone hydrochloride to conventional therapy, the pain from radiation-induced oral mucositis was effectively controlled (96.7% vs. 33.4%) and significantly improved patient eating (83.4% vs. 33.4%, *P* < 0.01)	—

Wu et al. [39]	Nasopharyngeal carcinoma	Conventional therapy (28) and oxycodone combined with conventional therapy (28)	∼5 mg	66∼70 Gy	Administered when the VAS score reached 4 or more in the 3rd week of radiotherapy	After adding oxycodone hydrochloride to conventional treatment, pain relief rate was significantly increased compared to the conventional therapy (92.8% vs. 32.1%, *P* < 0.05)	The adverse reactions can be relieved by symptomatic treatment. There were no withdrawal symptoms when medication was discontinued

Hisamitsu et al. [40]	Head and neck cancer	Mild pain (23) and moderate pain (20)	16.1 ± 0.8 mg vs 31.4 ± 4.4 mg	60 Gy	Administered when the pain reached a mild or moderate level	Between a cumulative dose of 25 and 50 Gy, opioid was introduced for pain control at a significantly Lower VAS in the mild pain group than the moderate pain group	No significant difference was found between the two groups (*P* > 0.05)

Cai and Liu [41]	Nasopharyngeal carcinoma	Moderate pain (11) and severe pain (24)	20–60 mg every 12 h	NA	Administered from the preparation period to the 2nd week of radiotherapy	The pain relief rate of both groups was 100%	The adverse reactions were relieved by symptomatic treatment

NA: not applicable; —: not mentioned.
